# Neuroprotective Effects of *Bifidobacterium breve* CCFM1067 in MPTP-Induced Mouse Models of Parkinson’s Disease

**DOI:** 10.3390/nu14214678

**Published:** 2022-11-04

**Authors:** Tiantian Li, Chuanqi Chu, Leilei Yu, Qixiao Zhai, Shunhe Wang, Jianxin Zhao, Hao Zhang, Wei Chen, Fengwei Tian

**Affiliations:** 1 State Key Laboratory of Food Science and Technology, School of Food Science and Technology, Jiangnan University, Wuxi 214122, China; 2 School of Food Science and Technology, Jiangnan University, Wuxi 214122, China; 3 National Engineering Research Center for Functional Food, Jiangnan University, Wuxi 214122, China; 4 Department of Child Health Care, The Affiliated Wuxi Maternity and Child Health Care Hospital of Nanjing Medical University, Wuxi 214002, China

**Keywords:** Parkinson’s disease, microbiota–gut–brain axis, *Bifidobacterium breve* 1067

## Abstract

There is mounting evidence that the microbiota–gut–brain axis (MGBA) is critical in the pathogenesis and progression of Parkinson’s disease (PD), suggesting that probiotic therapy restoring gut microecology may slow down disease progression. In this study, we examined the disease-alleviating effects of *Bifidobacterium breve* CCFM1067, orally administered for 5 weeks in a PD mouse model. Our study shows that supplementation with the probiotic *B. breve* CCFM1067 protected dopaminergic neurons and suppressed glial cell hyperactivation and neuroinflammation in PD mice. In addition, the antioxidant capacity of the central nervous system was enhanced and oxidative stress was alleviated. Moreover, *B. breve* CCFM1067 protected the blood–brain and intestinal barriers from damage in the MPTP-induced mouse model. The results of fecal microbiota analysis showed that *B. breve* CCFM1067 intervention could act on the MPTP-induced microecological imbalance in the intestinal microbiota, suppressing the number of pathogenic bacteria (*Escherichia-Shigella*) while increasing the number of beneficial bacteria (*Bifidobacterium* and *Akkermansia*) in PD mice. In addition, the increase in short chain fatty acids (acetic and butyric acids) may explain the anti-inflammatory action of *B. breve* CCFM1067 in the gut or brain of the MPTP-induced PD mouse model. In conclusion, we demonstrated that the probiotic *B. breve* CCFM1067, which can prevent or treat PD by modulating the gut–brain axis, can be utilized as a possible new oral supplement for PD therapy.

## 1. Introduction

By 2040, more than 17.5 million people will have Parkinson’s disease (PD), a frequent neurodegenerative disorder that affects the central nervous system (CNS) [1]. Despite this, there is presently no treatment that may prevent or cure PD. Two important pathological markers of PD are the degenerative death of midbrain nigrostriatal dopamine (DA) neurons and a significant reduction in striatal DA content, which in turn leads to motor deficits, such as stiffness, resting tremors, gait impairment, and bradykinesia in Parkinson’s disease [2,3,4]. People with PD usually have gastrointestinal problems and constipation years before they start having motor symptoms [5,6]. This suggests that gut disorders may be linked to the start of PD.

The gut microbiota may influence brain function and behavior through neurological, immunological, and endocrine pathways, establishing the microbiota–gut–brain axis (MGBA) [7,8]. Clinical investigations have shown that both the composition and abundance of gut bacteria and the produced metabolites are drastically altered in patients with PD compared to those in healthy patients [9]. One clinical study showed that the bacterial family Prevotellaceae is reduced, and beyond that, *Faecalibacterium prausnitzii* (a bacterial species that belongs to the phylum Firmicutes), as well as Lactobacillaceae and Enterococcaceae (two bacterial families that belong to the phylum Firmicutes) are reduced, and the bacterial family Enterobacteriaceae is more abundant in fecal samples from PD patients compared to matched controls [9]. Sampson et al. found that the motor impairment of germ-free PD-like mice worsened when they were administered the gut microbiota of patients with PD, demonstrating the role of gut microbes and short chain fatty acids (SCFAs) in α-synuclein-induced neurotoxicity and inflammation [10]. By transplanting the microbiota of young healthy mice into older PD mice, Marcus et al. showed that the age-related cognitive and behavioral impairments could be corrected, in part through altering the synthesis of SCFA and neurotransmitters [11]. The results of these studies indicate that one of the major contributors to PD is an imbalance of the gut microbiota.

Active bacteria that can colonize the human intestine are called probiotics, and when provided to the host at certain doses, they have beneficial effects on health. In a prior study, mixed probiotic formulations consisting of all of the following: *Bifidobacterium* (*B.*) *bifidum*, *B. longum*, and *Lactobacillus* (*L.*) *rhamnosus* GG were shown to preserve DA neurons and improve motor dyskinesia in a PD mouse model when administered daily for 16 weeks [12]. In double-blind randomized placebo-controlled studies (RCTs), probiotics have been found to have positive effects on individuals with PD, including alleviation of gastrointestinal symptoms such as stomach discomfort, distension, nausea, constipation, and spontaneous defecation [13,14,15,16,17]. Probiotic products including *L. acidophilus*, *B. bifidum*, *L. reuteri*, and *L. fermentum*, can improve movement disorders, suppress oxidative stress, and even regulate insulin and lipid metabolism when consumed by patients with PD for 12 weeks [13]. These studies show that patients with PD can benefit from probiotics. However, factors such as the specificity of the probiotic strain and conditions of the host are likely to change the final clinical results. Therefore, further research is required to validate the health-promoting benefits of specific probiotics.

In addition, multiple studies conducted in recent years have shown that *Bifidobacterium* probiotic formulations have a therapeutic effect on neurodegenerative disorders and may protect the CNS via the MGBA [18,19,20,21,22]. Treatment of an Alzheimer’s disease (AD) mouse model with the *B. breve* A1 strain (MCC1274) improved both cognitive decline and motor dysfunction [23]. Bedarf et al. observed that *B. breve* A1 oral treatment could inhibit aberrant alterations in hippocampal synaptic plasticity and relieve symptoms of situational fear disorder in mice with PD [24]. However, the mechanism and route through which *B. breve* A1 has a preventative effect on PD via the MGBA remains unknown [24]. While this study suggests that *B. breve* has a potential therapeutic effect in PD, no study has explained how *B. breve* probiotic supplementation affects PD through the MGBA.

The present study aimed to determine whether *B. breve* CCFM1067 has neuroprotective properties in an MPTP-PD mouse model. To further study the significance of the MGBA in the onset of PD, we also aimed to determine whether *B. breve* CCFM1067 might regulate the gut microbiota and alleviate PD motor dysfunction, resulting in a neuroprotective effect. Our findings suggest that the use of psychoactive probiotics such as *B. breve* CCFM1067 may prevent and alleviate PD.

## 2. Materials and Methods

### 2.1. Animals and Experimental Design

We used six-week-old male C57BL/6 mice acquired from Gempharmatech Co., Ltd., (Nanjing, China). Mice were kept in a controlled environment at 21–23 °C, with 50–60% humidity and a 12 h photoperiod. All the animals had unrestricted access to food and water throughout the feeding cycle. The Experimental Animal Ethics Committee of Jiangnan University approved all animal experiments (qualified number: JN. No20210630c1081020 [250]).

**Figure 1:**
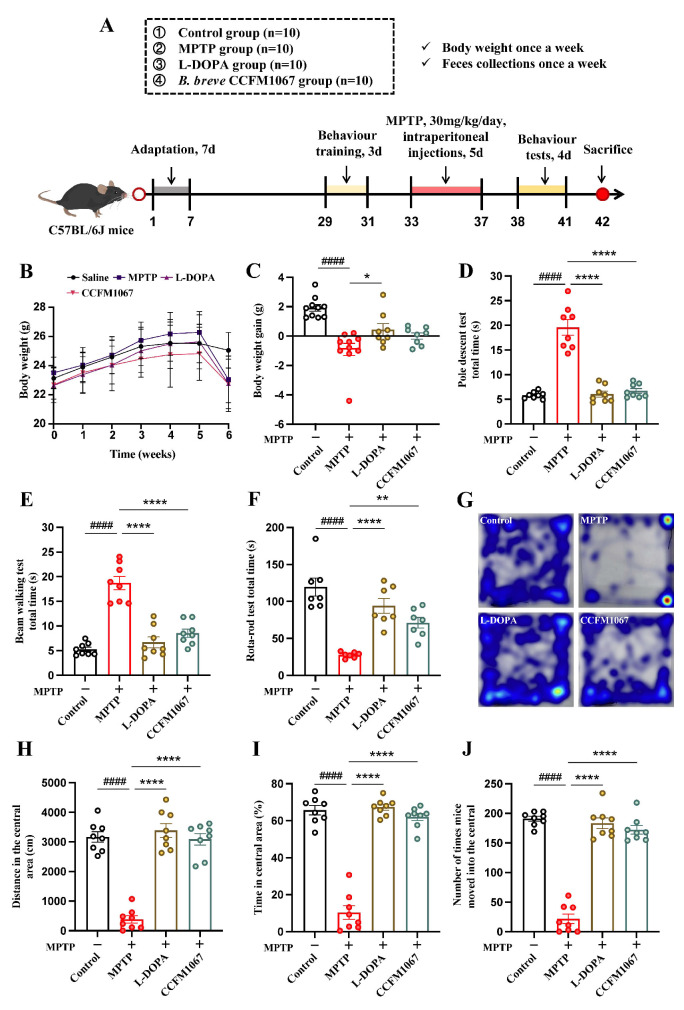
*Bifidobacterium breve* CCFM1067 alleviated MPTP-induced motor dysfunction in mice. (**A**) Timeline of animal experiments. (**B**) Changes in body weight of mice at six weeks (*n* = 10/group). (**C**) Increase in mouse body weight variation. (**D**) Pole descent test: total descent time as the evaluation indicator (*n* = 7/group). (**E**) Beam walking test: total walking time as the evaluation indicator (*n* = 7/group). (**F**) Rotarod test: total retention time as the evaluation indicator (*n* = 7/group). (**G**–**J**) Open field test (*n* = 8/group): (**G**) typical mouse tracking motion heat map for the four distinct mouse groups; (**H**) total distance traveled by PD mice in the center; (**I**) time spent in the open area/total active time spent by PD mice (%); (**J**) number of times mice moved into the central. Data are means with SEM; ^####^ *p* < 0.0001 vs. control group; * *p* < 0.05, ** *p* < 0.01, **** *p* < 0.0001 vs. MPTP group.

Figure 1A shows the animal experimentation protocol. Before beginning the experiments, the animals were allowed to acclimatize for one week. Forty mice were randomly divided into four groups of ten: control, MPTP, L-DOPA, and *B. breve* CCFM1067. Mice in the control, MPTP, and L-DOPA groups were administered 200 μL of saline daily from days 8 to 41. During days 33–37, with the exception of the control group, daily MPTP therapy was administered (30 mg/kg MPTP intraperitoneal injection; MedChemExpress, Shanghai, China). On days 33–41, the L-DOPA group received an oral gavage of L-DOPA (100 mg/kg; MedChemExpress) and benserazide (25 mg/kg; MedChemExpress) in saline. The *B. breve* CCFM1067 group received an oral gavage of *B. breve* CCFM1067 (10^9^ CFU/200 μL saline) daily on days 8–41, and MPTP treatment on days 33–37. All mice were weighed and feces were collected once a week. In addition, behavioral tests were performed on days 38–41. All mice were sacrificed on day 42 for further analysis.

### 2.2. Bifidobacterium breve CCFM1067 Preparation

The biological genesis of *Bifidobacterium breve* CCFM1067 is described in Supplementary Materials S1.1. *B. breve* CCFM1067 was incubated at 37 °C for 48 h after scribing onto MRS solid medium (containing 0.05% cysteine) to obtain a single colony. This single colony was then selected and inoculated in MRS liquid medium and cultured at 37 °C for 24 h for activation. Next, to obtain a *B. breve* CCFM1067 bacterial solution, the activation solution was inoculated at a concentration of 2% (*v*/*v*) with MRS liquid medium and incubated at 37 °C for 24 h. Then, the bacterial solution was centrifuged at 8000× *g* for 10 min to obtain *B. breve* CCFM1067. Suspensions were maintained at −80 °C.

### 2.3. Behavioral Tests for Motor Functions

Each mouse’s motor function was evaluated using four behavioral tests: pole test (PT), narrow-beam test (NBT), rotarod test (RTR), and open field test (OFT). Refer to Supplementary Materials S1.2 for specific behavioral experimental protocols. On days 29–31, all of the mice got behavioral training once a day. Tests of behavior were conducted 24 h following the final injection of MPTP on day 37.

### 2.4. Neurochemical and Biochemicall Analyses

Sample collection: Immediately after dissection, the striatum and midbrain were frozen at −80 °C for chemical analyses and mRNA expression experiments. For immunohistochemical staining, whole brains were removed and placed in a solution of 4% paraformaldehyde. Colon tissues were rinsed with saline and subjected to fast freezing in liquid nitrogen to preserve them at −80 °C for later use in chemical analysis and mRNA expression experiments. Mouse feces were collected from the colon for 16S rDNA gut microbiota analysis and SCFA extraction, and stored at −80 °C.

The concentrations of monoamines and their metabolites in the striatum were determined using High Performance Liquid Chromatography (HPLC) and fluorescence detection, as previously reported [25]. Following the manufacturer’s instructions, the levels of superoxide dismutase (SOD), glutathione (GSH), and catalase (CAT) in the brain substantia nigra (SN) supernatant were examined using analysis kits from the Nanjing Jincheng Bioengineering Institute. Inflammatory cytokines (TNF-α, IL-β, IL-6, and IL-10) were measured using enzyme-linked immunosorbent assay (ELISA) kits according to the manufacturer’s protocol (R&D Systems, Minnesota, USA). BDNF (brain-derived neurotrophic factor), GDNF (Glial cell line-derived neurotrophic factor), GFAP (glial fibrillary acidic protein) and Iba1 (ionized calcium binding adapter molecule 1), ZO-1 (zonula occludens-1), occludin, and claudin-1 were measured using ELISA kits purchased from SenBeiJia Biological Technology Co., Ltd., Nanjing, China. The short-chain fatty acid (SCFA) content in fresh mouse feces was measured using a TSQ 9000 GC-MS system (Thermo Scientific, Shanghai, China), and SCFAs were extracted from the feces with ether, as described previously [26].

### 2.5. Quantitative Real-Time Polymerase Chain Reaction (qRT-PCR)

Following the protocol described using total RNA extraction reagents (Vazyme, R401-01), RNA was extracted from the brain and intestinal tissue. Using the qPCR kit (Vazyme #R333, Nanjing, China), the extracted RNA was reverse-transcribed into cDNA. SYBR Green Supermix was used for quantitative PCR on a BioRad-CFX384 machine (Bio-Rad, California, USA). We used the cycle threshold (Ct) and normalized using the 2^−ΔΔCt^ technique relative to Gapdh to measure the relative expression of genes. In the Supplementary Materials S1.3, the RT-qPCR primers were detailed.

### 2.6. Immunohistochemistry

The paraformaldehyde-fixed and dehydrated brain tissue was embedded in paraffin and then processed according to standard paraffin section immunohistochemistry (IHC) experimental procedures. Specific procedures included the dewaxing of paraffin sections in water, antigen repair, endogenous peroxidase blocking, serum closure, and with mouse anti TH (1:500, EP1536Y, ab51253, Abcam, Cambridge, MA, USA) and secondary antibodies (goat anti-rabbit IgG, 1:200, GB23303, Servicebio, Wuhan, China) incubation. 3,3′-diaminobenzidine (DAB) staining was then used to observe SNpc TH-immunoreactive (TH-IR) neurons, next with re-staining of the nuclei, and then the sections were dehydrated and sealed. We used the Confocal laser scanning microscope equipment (ZEISS, Oberkochen, Germany) to digitize the stained slides, and then Image-J 1.53k was used for analysis.

### 2.7. Gut Microbial and Bioinformatics Analysis

The process for sequencing fecal 16S rRNA is discussed in full in Supplementary Materials S1.4. To analyze the raw sequencing data, we used the platform Quantitative Insights into Microbial Ecology (QIIME). Selected OTUs from the Greengenes 13.5 database was based on a nucleotide identity criteria of 97%. Species richness and distributional evenness among very uncommon OTUs were used to calculate the Observed, Shannon, and Simpson indices, which were used to evaluate the α-Diversity. β-Diversity was calculated using Bray–Curtis distances, displayed using principal component analysis (PCA) and principal coordinate analysis (PCoA), and differences were determined using permutational multivariate analysis of variance (perMANOVA). Microbiological biomarkers were separated using linear discriminant analysis (LDA) effect size (LEfSe) (Wilcoxon rank-sum test, *p* < 0.05 and log LDA > 4.0 was chosen as the criteria), which was shown as a taxonomic cladogram tree. Predicted functional pathways were annotated using the KEGG orthology, and the metagenomes of gut microbiota were computed from 16S rRNA sequences using the Phylogenetic Investigation of Communities by Reconstruction of Unobserved States (PICRUSt) method. Using the Wekemo Bioincloud (https://www.bioincloud.tech (accessed on 20 June 2022)), the aforementioned studies were conducted. The network of associations was constructed using Spearman’s correlation coefficients and the OmicStudio tools (https://www.omicstudio.cn/tool (accessed on 29 June 2022)). Unless otherwise stated, suggested settings were always utilized.

### 2.8. Statistical Analysis

The statistical analyses were conducted using the GraphPad Prism 9.1 software. Data are presented as the mean ± SEM. The experimental data were checked for conformance to a normal distribution using the D’Agostino–Pearson test. If the datasets were normally distributed, Student’s unpaired *t*-test or one-way analysis of variance (ANOVA) with Dunnett’s test was used to compare two or multiple datasets, respectively. Conversely, if the datasets were not normally distributed, the Mann–Whitney test or Kruskal–Wallis with Dunn’s test was conducted to compare two or multiple datasets, respectively.

## 3. Results

### 3.1. B. breve CCFM1067 Improves MPTP-Induced Motor Impairments

The experimental design is shown in Figure 1A. Mice in the MPTP group started to lose weight on day 35, during the development of the PD model (Figure 1B). As shown in Figure 1C, L-DOPA therapy significantly lowered weight loss in MPTP-induced mice, whereas *B. breve* CCFM1067 treatment reduced weight loss marginally, but not significantly (*p* > 0.05). Motor function tests, including PT, NBT, RTR, and OFT, were used to assess the effectiveness of *B. breve* CCFM1067 on the locomotor coordination of PD mice. Compared to the control group, mice in the MPTP group displayed significant motor impairments, including decreased motor agility (Figure 1D, T (14) = 8.625, *p* < 0.0001), motor balance (Figure 1E, T (14) = 9.378, *p* < 0.0001), and motor coordination (Figure 1F, T (12) = 7.487, *p* < 0.0001). Both L-DOPA and *B. breve* CCFM1067 therapies substantially reduced MPTP-induced motor impairments in the PT (Figure 1D, F (2,21) = 56.94, *p* < 0.0001), NBT (Figure 1E, F (2,21) = 33.72, *p* < 0.0001), and RTR (Figure 1F, F (2,18) = 21.99, *p* < 0.0001). The OFT results indicated that *B. breve* CCFM1067 showed the same tendency as that of the PT, NBT, and RTR tests (Figure 1G–J). MPTP-induced PD mice moved less towards the center (Figure 1G, T (12) = 12.88, *p* < 0.0001), where they had a shorter residence time (Figure 1H, T (14) = 12.53, *p* < 0.0001) and fewer entries (Figure 1J, T (14) = 18.44, *p* < 0.0001) than those of normal control mice, which could be significantly ameliorated by L-DOPA and *B. breve* CCFM1067 treatment (all *p* < 0.0001). Collectively, these results suggest that therapy with *B. breve* CCFM1067 mitigates MPTP-induced motor impairment.

### 3.2. B. breve CCFM1067 Alleviates MPTP-Induced Neuropathological Alterations

**Figure 2:**
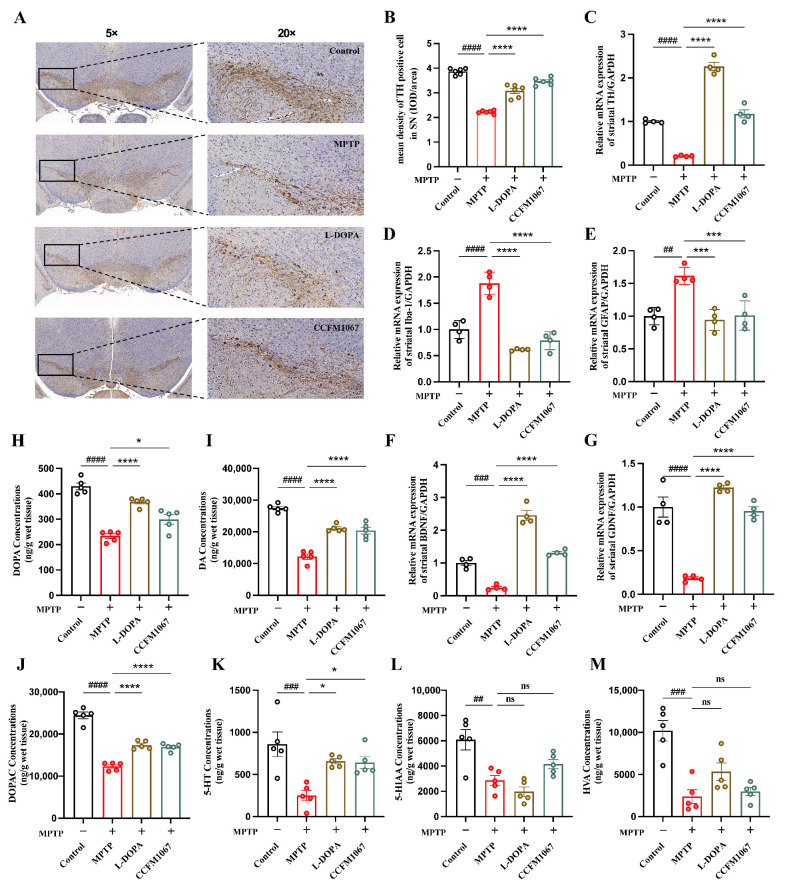
*B. breve* CCFM1067 alleviated MPTP-induced neuropathologic changes and glial activation, restored the MPTP-induced reduction of the levels of striatal neurotransmitters. (**A**) Images of immunohistochemically stained cells positive for tyrosine hydroxylase (TH) in the SN (Under 5× and 20× magnification, one typical picture from each group). (**B**) The mean density of TH positive cells in SN in each group (*n* = 3/group). The mRNA expressions of TH (**C**), Iba-1 (**D**) and GFAP (**E**); BDNF (**F**) and GDNF (**G**) in the striatum (*n* = 4/group). The concentrations of neurotransmitters in the striatum: L-DOPA (**H**), DA, dopamine (**I**), DOPAC, 3,4-dihydroxyphenylacetic acid (**J**), 5-HT, 5-hydroxytryptamine (**K**), 5-HIAA, 5-hydroxyindoleacetic acid (**L**), and HVA, homovanillic acid (**M**) (*n* = 5/group). Data are means with SEM; ^##^ *p* < 0.01, ^###^ *p* < 0.001, ^####^ *p* < 0.0001 vs. control group; * *p* < 0.05, *** *p* < 0.001, **** *p* < 0.0001 vs. MPTP group, “ns” means there was no noticeable difference between the groups.

The percentage of mice with positive TH cell counts in the SN is shown in Figure 2B. A comparison with the control group revealed that MPTP dramatically decreased TH expression in the SN of mice (T (10) = 27.92, *p* < 0.0001). TH expression was reduced with L-DOPA and *B. breve* CCFM1067 treatment, with *B. breve* CCFM1067 (Figure 2B, F (2,15) = 77.15, *p* < 0.0001) being the most efficient (Figure 2B). These results demonstrate that *B. breve* CCFM1067 alleviated MPTP-induced neuropathological alterations.

We studied whether *B. breve* CCFM1067 affects glial reactivity in PD mice. RT-qPCR was used to quantify the gene expression levels of glial markers (Iba-1 and GFAP) and neurotrophic factors (BDNF and GDNF) in the striatum. Both striatal Iba-1 (Figure 2D, T (6) = 6.405, *p* = 0.0007) and GFAP (Figure 2E, T (6) = 6.619, *p* = 0.0006) mRNA expression levels were markedly increased, while those of BDNF (Figure 2F, T (6) = 8.863, *p* = 0.0001) and GDNF (Figure 2G, T (6) = 6.988, *p* = 0.0004) were significantly reduced in the MPTP group. However, *B. breve* CCFM1067 markedly attenuated the mRNA expression of striatal Iba-1 (F (2,9) = 73.33, *p* < 0.0001) and GFAP (F (2,9) = 17.72, *p* = 0.0017), greatly increased the mRNA expression of striatal BDNF (F (2,9) = 143.2, *p* < 0.0001) and GDNF (F (2,9) = 267.2, *p* < 0.0001), and restored their normal levels (Figure 2D–G). In addition, we measured protein expression levels of BDNF, GDNF, GFAP, and Iba-1 in the striatum. ELISA showed that MPTP induction significantly decreased the BDNF (T (6) = 9.244, *p* < 0.0001) and GDNF (T (6) = 8.333, *p* = 0.0002) levels, and markedly increased the GFAP (T (6) = 10.61, *p* < 0.0001) and Iba-1 (T (6) = 7.915, *p* = 0.0002) levels in the striatum of the MPTP group compared with that of the control group (Figure S1A–D). *B. breve* CCFM1067 intake successfully alleviated elevated striatal GFAP (F (2,9) = 19.66, *p* = 0.0024) and Iba-1 (F (2,9) = 21.34, *p* = 0.0015) levels, as well as reduced BDNF (F (2,9) = 39.32, *p* = 0.0005) and GDNF (F (2,9) = 26.74, *p* = 0.0013) levels in MPTP-treated mice (Figure S1A–D). These findings show that the reduction of glial activation and boosting of neurotrophins are the two mechanisms through which *B. breve* CCFM1067 protects the striatum.

To understand the mechanism underlying the observed behavioral improvement, we analyzed the effects of *B. breve* CCFM1067 on neurotransmitters and their metabolites in PD mouse striata. HPLC experiments demonstrated that MPTP therapy drastically decreased the levels of DOPA (Figure 2H, T (8) = 12.07, *p* < 0.0001), DA (Figure 2I, T (8) = 15.54, *p* < 0.0001), DOPAC (Figure 2J, T (8) = 13.47, *p* < 0.0001), 5-HT (Figure 2K, T (8) = 3.836, *p* = 0.0050), 5-HIAA (Figure 2L, T (8) = 3.594, *p* = 0.0070), and HVA (Figure 2M, T (8) = 5.336, *p* = 0.0007) in the striatum. Supplementation with *B. breve* CCFM1067 enhanced striatal DOPA (F (2,12) = 27.45, *p* = 0.0067) and 5-HT (F (2,12) = 15.94, *p* = 0.0009) levels, and dramatically increased those of DA (F (2,12) = 36.39, *p* < 0.0001) and DOPAC (F (2,12) = 45.03, *p* < 0.0001) in mice with PD (Figure 2H–K). However, the decrease in 5-HIAA and HVA (Figure 2L,M; *p* > 0.05) caused by MPTP was not affected by L-DOPA or *B. breve* CCFM1067 treatment. Collectively, these results indicate that *B. breve* CCFM1067 reduced the neuropathological changes and glial activation caused by MPTP and restored the normal levels of neurotransmitters in PD mice.

### 3.3. B. breve CCFM1067 Increases Antioxidant Levels and Reduces MPTP-Induced Neuroinflammation

**Figure 3:**
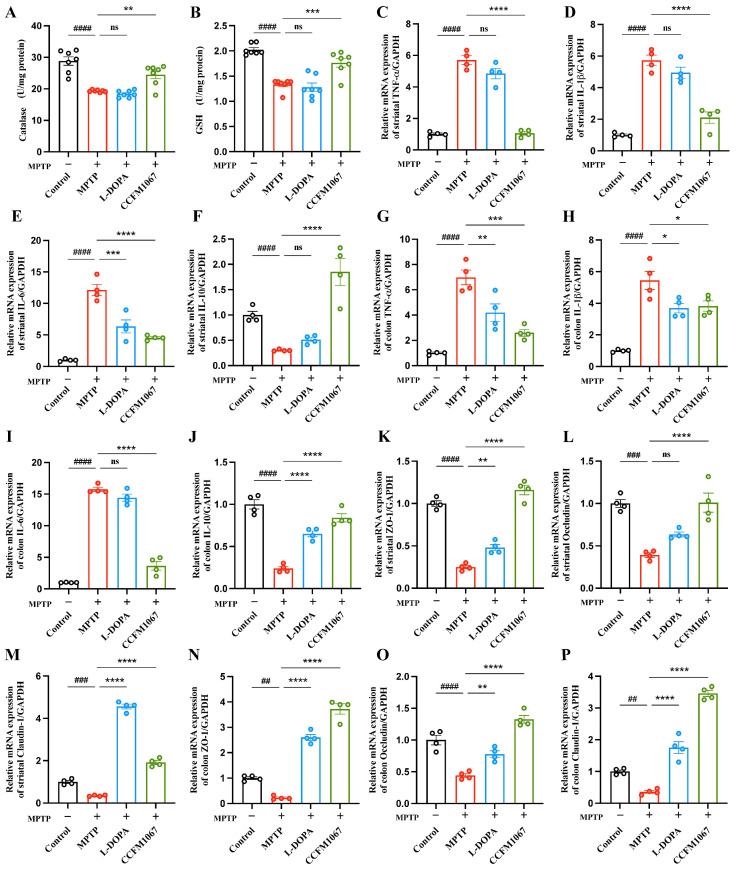
*B. breve* CCFM1067 reduced MPTP-induced oxidative stress, suppressed the generation of pro-inflammatory molecules in the SN and colon, and alleviated intestinal barrier damage. The antioxidant activities of catalase (**A**) and GSH (**B**) in the midbrain (U/mg protein) (*n* = 7/group). (**C**–**J**) The mRNA expression of TNF-α, IL-1β, IL-6 and IL-10 in the mouse midbrain and colon (*n* = 4/group). (**K**–**P**) The mRNA expression of ZO-1, occludin, and claudin-1 in the midbrain and colon of mice (*n* = 4/group). Data are means with SEM; ^##^ *p* < 0.01, ^###^ *p* < 0.001, ^####^ *p* < 0.0001 vs. control group; * *p* < 0.05, ** *p* < 0.01, *** *p* < 0.001, **** *p* < 0.0001 vs. MPTP group, “ns” means there was no noticeable difference between the groups.

We examined the midbrain antioxidant levels in each group of mice. Catalase levels were substantially decreased by MPTP treatment (Figure 3A, T (12) = 7.080, *p* < 0.0001; Figure 3B, T (12) = 11.55, *p* < 0.0001), whereas *B. breve* CCFM1067 intake reversed these effects (Figure 3A, F (2,18) = 18.09, *p* = 0.0003; Figure 3B, F (2,18) = 13.05, *p* = 0.0012). The L-DOPA intervention group showed no effect on the MPTP-induced reduction in catalase and GSH levels (Figure 3A,B, *p* > 0.05). In the experimental groups, none of the midbrain SOD levels varied appreciably.

Growing evidence suggests that gut inflammation contributes to the onset of PD. Cytokine measurements in the striatum and colon were performed using RT-qPCR to determine the expression levels of pro- and anti-inflammatory cytokines. Our findings revealed that MPTP induction caused TNF-α (Figure 3C, T (6) = 16.30, *p* < 0.0001), IL-1β (Figure 3D, T (6) = 13.81, *p* < 0.0001), and IL-6 (Figure 3E, T (6) = 12.26, *p* < 0.0001) levels to rise significantly in the striatum. Ingestion of *B. breve* CCFM1067 reduced the increase in striatal TNF-α (Figure 3C, F (2,9) = 91.49, *p* < 0.0001), IL-1β (Figure 3D, F (2,9) = 29.53, *p* < 0.0001), and IL-6 (Figure 3E, F (2,9) = 24.61, *p* = 0.0002). *B. breve* CCFM1067 treatment also significantly increased IL-10 levels (Figure 3F, F (2,9) = 28.76, *p* = 0.0001), which were significantly decreased by MPTP induction. The expression of these three pro-inflammatory genes was also elevated in the MPTP group (Figure 3G–I: TNF-α, T (6) = 10.18, *p* < 0.0001; IL-1β, T (6) = 7.762, *p* = 0.0002; IL-6, T (6) = 44.71, *p* < 0.0001), consistent with the findings in the striatum. In contrast, *B. breve* CCFM1067 intervention significantly suppressed the expression of inflammatory genes (Figure 3G–I: TNF-α, F (2,9) = 16.52, *p* = 0.0006; IL-1β, F (2,9) = 5.522, *p* = 0.0396; IL-6, F (2,9) = 148.9, *p* < 0.0001). Besides, the ELISA analysis of these 4 proteins was also remarkably raised by MPTP administration in the striatum (TNF-α, T (6) = 6.860, *p* = 0.0005; IL-1β, T (6) = 9.800, *p* < 0.0001; IL-6, T (6) = 10.95, *p* < 0.0001) and the colon (TNF-α, T (6) = 12.3, *p* < 0.0001; IL-1β, T (6) = 15.07, *p* < 0.0001; IL-6, T (6) = 11.12, *p* < 0.0001) (Figure S2A–H), which was suppressed by *B. breve* CCFM1067 treatment in the striatum (TNF-α, F (2,9) = 20.99, *p* = 0.0002; IL-1β, F (2,9) = 21.76, *p* = 0.0396; IL-6, F (2,9) = 29.11, *p* < 0.0001) and the colon (TNF-α, F (2,9) = 34.1, *p* < 0.0001; IL-1β, F (2,9) = 50.34, *p* < 0.0001; IL-6, F (2,9) = 21.02, *p* = 0.0003) (Figure S2A–H). Overall, *B. breve* CCFM1067 suppressed pro-inflammatory gene expression and protein expression, decreased inflammation in the brain and colon, and boosted anti-inflammatory capabilities in PD mice.

### 3.4. B. breve CCFM1067 Treatment Helps Improve Blood–Brain and Intestinal Barrier Damage

The mRNA expression of three key tight junction proteins was evaluated in the striatum and colon of mice to ascertain whether neuroinflammation was linked to blood–brain barrier (BBB) leakage. As displayed in Figure 3K–P, MPTP administration in PD mice led to a substantial decrease in striatal ZO-1 (Figure 3K, T (6) = 19.82, *p* < 0.0001), *occludin* (Figure 3L, T (6) = 11.41, *p* < 0.0001), claudin-1 (Figure 3M, T (6) = 9.672, *p* < 0.0001), and colon *ZO-1* (Figure 3N, T (6) = 12.27, *p* < 0.0001), occludin (Figure 3O, T (6) = 6.899, *p* = 0.0005), and *claudin-1* (Figure 3P, T (6) = 9.862, *p* < 0.0001) mRNA levels. In contrast, *B. breve* CCFM1067 treatment greatly enhanced the expression of these genes in the striatum and colon compared to that in the MPTP group, and at even higher levels than that in the control group. Moreover, the ELISA analysis of these three proteins validated its reduced expression in the MPTP group (striatum, ZO-1, T (6) = 9.204, *p* < 0.0001; occludin, T (6) = 9.419, *p* < 0.0001; claudin-1, T (6) = 6.328, *p* = 0.0007; colon, ZO-1, T (6) = 4.383, *p* = 0.0047; occludin, T (6) = 4.755, *p* < 0.0001; claudin-1, T (6) = 6.913, *p* = 0.0005) and its increased expression in the *B. breve* CCFM1067 group (striatum, ZO-1, F (2,9) = 29.24, *p* < 0.0001; occludin, F (2,9) = 23.94, *p* = 0.0004; claudin-1, F (2,9) = 24.76, *p* = 0.0001; colon, ZO-1, F (2,9) = 26.80, *p* = 0.0001; occludin, F (2,9) = 14.40, *p* = 0.0010; claudin-1, F (2,9) = 4.646, *p* < 0.026) (Figure S3A–F). These results indicate that *B. breve* CCFM1067 protects against BBB damage and intestinal barrier dysfunction in an MPTP-induced mouse model.

### 3.5. B. breve CCFM1067 Ameliorated the Dysbiosis of the Mouse Gut Microbiota of MPTP-Induced

**Figure 4:**
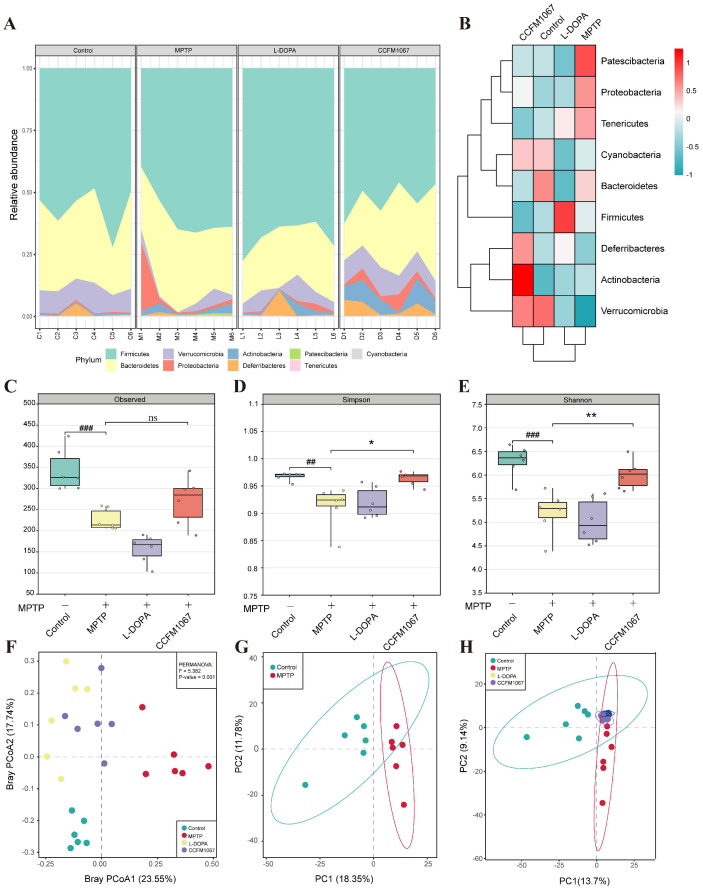
Phylum−level changes and gut microbiota diversity analysis among the control, MPTP, L−DOPA, and *B. breve* CCFM1067 groups (**A**) Stacked bar plots representing the relative abundance of phyla in individual samples from the four different groups. (**B**) Heat map of the relative abundance of nine bacterial phyla. (**C**–**E**) α−diversity analysis: Observed, Shannon, and Simpson indexes. (**F**) PCoA of β−diversity. The Bray–Curtis matrix, analyzed with PERMANOVA, was used to evaluate the differences between groups. The axes are labeled with the contribution vectors (PCoA1 and PCoA2) of the main components, and mouse samples are color-coded to distinguish across groups. (**G**) Principal component analysis (PCA) of β−diversity between the control and MPTP−treated groups. (**H**) PCA of β−diversity among the four groups. Data are means with SEM; ^##^ *p* < 0.01, ^###^ *p* < 0.001 compared with the Control group; * *p* < 0.05, ** *p* < 0.01 vs. MPTP group, “ns” means there was no noticeable difference between the groups.

To determine how *B. breve* CCFM1067 protects the MPTP-induced PD mouse model by modifying the microbial community structure, we sequenced 16S rRNA from feces collected six days after MPTP treatment. Nine bacterial phyla dominated the gut microbiota in all four groups (Figure 4A). As shown in Figure 4B, the phyla Patescibacteria, Proteobacteria, and Tenericutes showed a considerably increased abundance in PD mice, while the phyla Verrucomicrobia, Cyanobacteria, and Deferribacteres had a significantly decreased abundance (vs. control). Mice with PD showed significant microbial changes, which *B. breve* CCFM1067 was shown to correct.

According to the α-diversity analysis, the microbiota of PD mice exhibited a lower Observed index (Figure 4C, T (10) = 5.147, *p* = 0.0004), which is closely linked to microbial richness, and lower Shannon and Simpson index values (Figure 4D, T (10) = 3.468, *p* = 0.006; Figure 4E, T (10) = 4.738, *p* = 0.0008), which are closely correlated with microbial diversity (vs. control). Treatment with *B. breve* CCFM1067 considerably increased the Shannon (Figure 4E, F (2,15) = 5.858, *p* = 0.0122) and Simpson (Figure 4E, F (2,15) = 9.485, *p* = 0.0081) values (vs. MPTP), whereas L-DOPA therapy had no effect on these (all *p* > 0.05, vs. MPTP). These findings show that *B. breve* CCFM1067 treatment may reverse the reduction in microbial community richness and diversity in PD mice. Moreover, the β-diversity analysis indicated that the gut microbial community of the MPTP group was considerably different from that of the control group, whereas that of the *B. breve* CCFM1067 and L-DOPA groups was similar to that of the control (Figure 4F, PERMANOVA: F = 5.382, *p* = 0.001). The results of the principal component analysis (PCA) were the same as that above (Figure 4G,H). The findings on intestinal microbiota diversity and structure demonstrate that therapy with *B. breve* CCFM1067 effectively controlled alterations in the gut microbiota of MPTP-treated mice.

**Figure 5:**
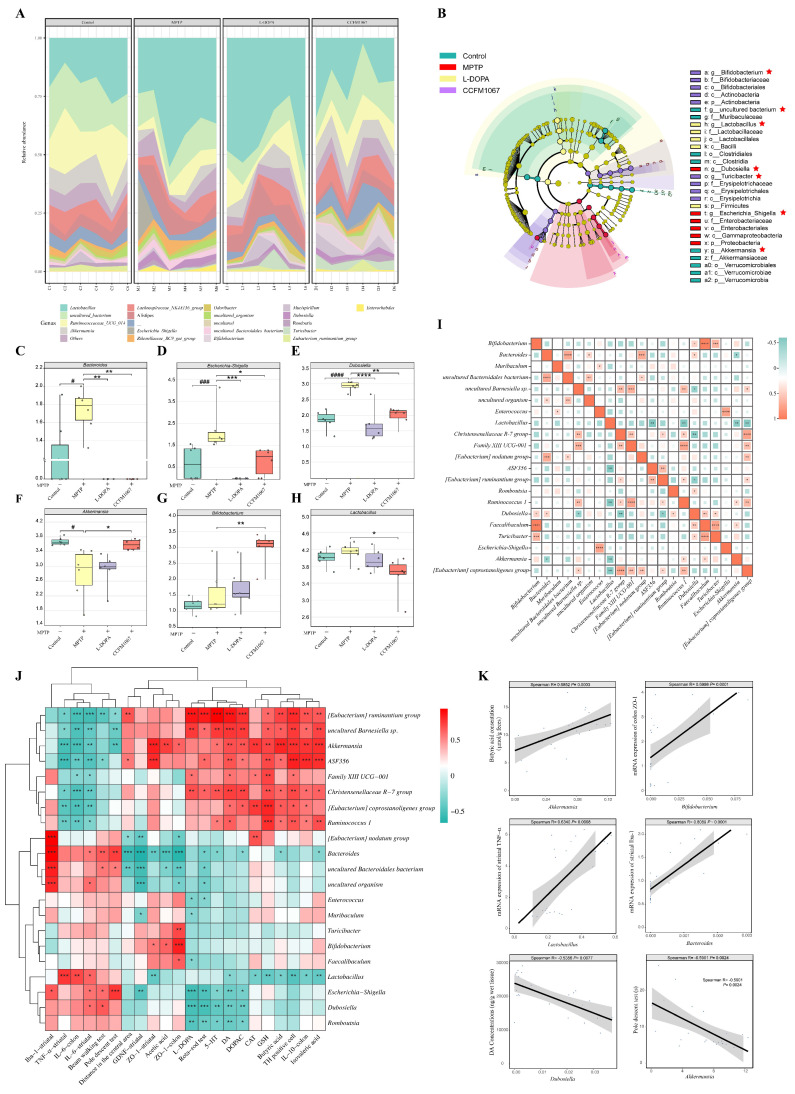
*B. breve* CCFM1067 intervenes to regulate MPTP−induced changes in the gut microbial composition of PD mice. (**A**) Stacked bar plots representing the relative abundance of genera in individual samples from the four different groups. (**B**) LEfSe analysis of the control, MPTP, L−DOPA, and *B. breve* CCFM1067 groups. Species without significant differences are colored uniformly in yellow, and the differential species biomarker follows group coloration. The figure shows the species with significant differences in abundance between groups at *p* < 0.05 and log LDA > 4.0. (**C**–**H**) Six genus levels are shown in the figure, namely *Bacteroides* (**C**), *Escherichia*−*Shigella* (**D**), *Dubosiella* (**E**), *Akkermansia* (**F**), *Bifidobacterium* (**G**) and *Lactobacillus* (**H**). The data in the figure are normalized by log_10_. (**I**) Correlation analysis of the total significantly different 21 genera between the four groups. The color and size of the square represent the degree of correlation determined using two-tailed Spearman’s analysis. (**J**) Spearman’s correlation analysis between 22 other experimentally determined PD biomarkers and the 21 different genera. Red indicates positive correlation, green negative, and color intensity indicates the strength of the correlation. (**K**) Positive correlation between fecal *Akkermansia* abundance and the level of butyric acid in mouse feces; positive correlation between fecal *Bifidobacterium* abundance and the mRNA expression of ZO-1 in the colon of mice; positive correlation between fecal *Lactobacillus* abundance and the mRNA expression of TNF-α in the midbrain of mice; positive correlation between fecal *Bacteroides* abundance and the mRNA expression of Iba-1 in the mouse striatum; the amount of DA in the mouse striatum was inversely proportional to the amount of *Dubosiella* in the feces; the abundance of fecal *Akkermansia* was inversely proportional to the amount of time required to complete the pole descent test. Spearman’s rho correlation coefficient and the regression line are shown. Data are means with SEM; ^#^ *p* < 0.05, ^###^ *p* < 0.001, ^####^ *p* < 0.0001, compared with the control group; * *p* < 0.05, ** *p* < 0.01, *** *p* < 0.001, **** *p* < 0.0001 vs. MPTP group, “ns” means there was no noticeable difference between the groups.

We evaluated the relative abundance of microorganisms at the taxonomic genus level in various groups (Figure 5A) and screened seven differential genera in the control, MPTP, L-DOPA, and *B. breve* CCFM1067 groups using LEfSe analysis (Figure 5B). In addition, the results of LEfSe analysis between the control and MPTP groups, the MPTP and *B. breve* CCFM1067 groups, and the MPTP and L-DOPA groups for each two groups were shown in Figure S4. MPTP-induced mice exhibited a lower relative abundance of *Akkermansia* (Figure 5F, T (10) = 3.082, *p* = 0.0116) and a higher relative abundance of *Bacteroides* (Figure 5C, T (10) = 2.796, *p* = 0.0189), *Escherichia-Shigella* (Figure 5D, T (10) = 5.165, *p* = 0.0004), and *Dubosiella* (Figure 5E, T (9) = 8.641, *p* < 0.0001) compared to those in the control group. Nonetheless, *B. breve* CCFM1067 intervention significantly restored the abundance of the above-mentioned bacterial genera, suggesting that *B. breve* CCFM1067 intervention could reverse MPTP-induced abnormal microbial composition in mice.

Twenty-one genera exhibited significant changes in bacterial abundance when examined using the univariate approach for the factorial Kruskal–Wallis test (*p* < 0.05, FDR < 0.05). These 21 genera were used to create a heatmap of gut microbial marker correlations (Figure 5I). Because the gut microbiota interacts to maintain a dynamic equilibrium, examining the interactions between various genera may help us understand how these species affect the onset of PD. To further show the impact of microbiota on mice treated with MPTP, correlations between the abundance of bacterial genera and other experimental outcomes were analyzed (Figure 5I,J). These findings suggest that *B. breve* CCFM1067 alleviates microbiota dysbiosis in mice with PD, highlighting the importance of microbial dysbiosis in PD progression.

### 3.6. Functional Predictions Suggested That B. breve CCFM1067 May Modify Functional Modules of the Gut Microbiota

**Figure 6:**
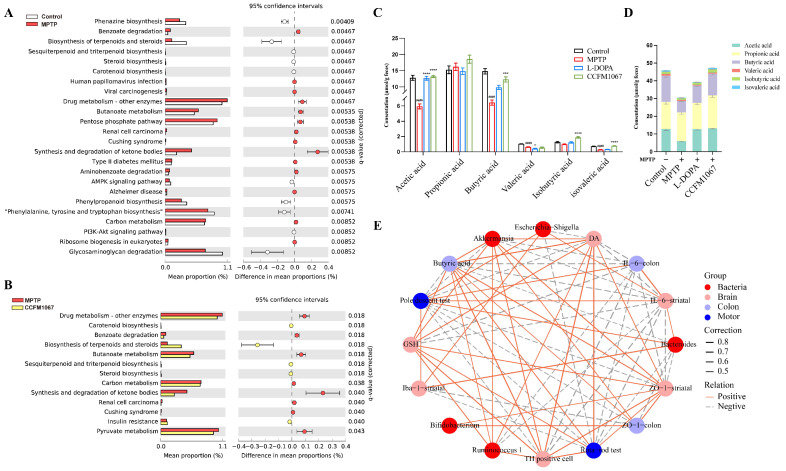
*B. breve* CCFM1067 treatment affects gut microbial functions in MPTP-induced PD mice, which may be mediated by microbial metabolites. (**A**) Differences in predicted functions between control and MPTP groups in the Kyoto Encyclopedia of Genes and Genomes (KEGG) pathways at level 3 (Welch’s *t* test, two-sided, Storey FDR *q* < 0.01). (**B**) Differences in predicted functions between *B. breve* CCFM1067 and MPTP groups in the KEGG pathways at level 3 (Storey FDR *q* < 0.05). (**C**) Concentration (µmol/g feces) of SCFAs. (**D**) Total SCFAs: the amount of acetic, propionic, isobutyric, butyric, isovaleric, and valeric acids add up to the total SCFAs (*n* = 5/group). (**E**) A network of correlations between different experimental findings in the microbiota–gut–brain axis. The different node colors show different classifications, the red, pink, purple, and blue nodes correspond to the experimental results regarding the gut microbiota, brain, colon, and motor function of the mice. Negative Spearman correlation coefficients are represented by gray, dashed lines (R < −0.5), whereas positive correlation coefficients are shown with solid orange lines (R > 0.5). Spearman correlation coefficient values below −0.5 (negative correlation) are indicated with gray dotted lines, and coefficient values above 0.5 (positive correlation) are indicated with solid orange lines. The strength of the correlation is indicated by the thickness of the line. Data are means with SEM; unpaired Student’s *t*-test ^####^ *p* < 0.0001, compared with the control group; one-way ANOVA with Dunnett’s test for multiple comparisons between MPTP, L-DOPA, and *B. breve* CCFM1067 groups, * *p* < 0.05, *** *p* < 0.001, **** *p* < 0.0001 vs. MPTP group, “ns” means there was no noticeable difference between the groups.

We conducted a Kyoto Encyclopedia of Genes and Genomes (KEGG) pathway enrichment analysis to better understand the functions of these significantly altered microorganisms. Twenty-four KEGG (level 3) pathways were differentially enriched between the control and MPTP groups (*p* < 0.01, with Q value < 0.01) (Figure 6A), and MPTP increased the pathways involved in benzoate degradation, human papillomavirus infection, viral carcinogenesis, drug-metabolizing enzymes, butanoate metabolism, pentose phosphate, renal cell carcinoma, Cushing syndrome, carbon metabolism, and ribosome biogenesis in eukaryotes (Figure 6A). Thirteen KEGG (level 3) pathways were differentially enriched between *B. breve* CCFM1067 and MPTP (*p* < 0.05, Q value < 0.05) (Figure 6B), and *B. breve* CCFM1067 increased pathways involved in the biosynthesis of carotenoids, terpenoids and steroids, sesquiterpenoids and triterpenoids, steroids, and insulin resistance (Figure 6B). Differences in predicted functions between L-DOPA and MPTP groups at the KEGG pathways at level 3 were shown in Figure S5 (*p* < 0.05, Q value < 0.05). In addition, the Spearman correlation analysis between 21 different genera and 32 different KEGG pathways showed that many different genera were significantly correlated with different KEGG pathways (Figure S6).

### 3.7. Correlations Support the Involvement of the MGBA in the MPTP-Treated Mouse Model

We measured the levels of SCFAs in intestinal contents (Figure 6C,D). The levels of acetic acid, butyric acid, valeric acid, and isovaleric acid (all *p* < 0.001) were significantly lower in the MPTP group compared to those in the control group. In contrast, *B. breve* CCFM1067 greatly increased the concentration of SCFAs (Figure 6C: acetic acid, F (2, 21) = 87.78, *p* < 0.0001; butyric acid, F (2, 24) = 11.08, *p* = 0.0002; isobutyric acid, F (2, 24) = 26.18, *p* < 0.0001; isovaleric acid, F (2, 24) = 159.0, *p* < 0.0001) with the exception of propionic acid, the level of which was not affected by any of the treatments (Figure 6C). Therefore, *B. breve* CCFM1067 affected the MPTP-induced SCFA decline in the mouse colon.

We conducted a correlation analysis between key experimental data to gain a deeper understanding of the processes involved in the MGBA in the pathogenesis of PD mice and the preventive benefits of *B. breve* CCFM1067 therapy. The relative abundances of *Bifidobacterium*, *Bacteroides*, *Ruminococcus-1*, *Escherichia-Shigella*, and *Akkermansia* represented the microbiota changes in the different groups. The total time in the pole test and total retention time on the rotarod test were selected as indicators for changes in motor ability. To illustrate the changes in the brain, the number of TH-positive cells in the SN; the expression of *Iba-1*, *IL-6*, and *ZO-1* in the striatum; the levels of DA in the striatum; and the level of GSH in the midbrain were assessed. We also chose the level of butyric acid in colon feces and the expression of *IL-6* and *ZO-1* in the colon as important features of the gut.

Surprisingly, we discovered that the majority of associations between these markers were statistically significant. These correlation data jointly suggest the participation of the MGBA in the development of the MPTP-induced PD mouse model, as well as the protective effects of *B. breve* CCFM1067 therapy in this model.

## 4. Discussion

To test whether *B. breve* CCFM1067 helps regulate the intestinal microbiota and assess its neuroprotective potential, we used a subacute PD mouse model obtained through intraperitoneal MPTP injection. Our results show that *B. breve* CCFM1067 intake exhibited neuroprotective effects, significantly alleviating motor deficits in PD mice (Figure 1D–J), protecting dopaminergic neurons in the midbrain (Figure 2A,B), and increasing the levels of striatal neurotransmitters (Figure 2H–M). Ingestion of *B. breve* CCFM1067 also enhanced endogenous neuroprotective factors in the brains of mice (Figure 2F,G) and alleviated MPTP-induced neuroinflammation (Figure 3C–E) and oxidative stress (Figure 3A,B). Ingestion of *B. breve* CCFM1067 restored the BBB and the impaired intestinal barrier (Figure 3K–P), alleviated dysbiosis of the intestinal flora (Figure 4 and Figure 5), and increased the SCFA content in the feces of PD mice (Figure 6). Therefore, we herein demonstrate that a particular strain of *Bifidobacterium breve* CCFM1067, may ameliorate PD-related symptoms through the MGBA.

DA in the brain works in tandem with the glutamate system to control motor and cognitive functions of the body [27]. DA synthesis is limited by the rate-limiting enzyme TH, and motor symptoms of PD emerge when TH levels drop below a particular threshold [28,29]. According to histopathological staining findings, ingestion of *B. breve* CCFM1067 reduced the death of dopaminergic neurons in the SN. Liao et al. demonstrated that probiotics protect neurons by preventing a decline in TH in dopaminergic neurons and boosting DA expression [7]. The neurotransmitters DA and 5-HT play essential roles in the brain [30]. In agreement with the findings of other studies, we found that *B. breve* CCFM1067 significantly enhanced the levels of neurotransmitters, such as DA, DOPAC, and 5-HT, in the striatum of PD mice. Neurotrophic factors, such as BDNF and GDNF, have been shown to protect dopaminergic neurons in PD mice by preventing neuronal death [31,32]. BDNF may modulate the neuroinflammatory response through the m-BDNF-tropomyosin receptor kinase B (TrkB) pathway, in addition to its role in maintaining neuronal survival [33,34,35]. In our study, after *B. breve* CCFM1067 intervention, PD mice exhibited a significant improvement in motor dysfunction, a significant reduction in dopaminergic neuron loss, increased neurotransmitter levels in the striatum, and restored BDN*F* and GDNF expression, suggesting *B. breve* CCFM1067 has a neuroprotective effect against MPTP-induced PD.

ROS are regarded as a major threat to bodily health, and their activation can perturb the redox equilibrium [36]. An increasing body of research indicates that ROS generation exacerbates α-synuclein misfolding in the brain, resulting in PD and other synaptic proteinopathies [37,38]. In clinical studies, probiotics have been shown to lower the development of α-synuclein aggregates by lowering ROS levels, thereby improving the condition of patients with PD [37,39]. In our study, MPTP-treated mice showed considerably lower CAT and GSH levels in the midbrain. Restoration of CAT and GSH antioxidant capabilities under MPTP toxicity after the intake of *B. breve* CCFM1067 is shown in Figure 3, indicating that this supplement partially attenuated MPTP-induced superoxide toxicity by increasing antioxidant levels. GSH levels in health and disease can provide important information regarding neuronal health [40,41]. Although GSH is not the sole antioxidant involved in PD, it seems to relate with disease severity because GSH depletion starts before other PD symptoms, including diminished complex I activity in the mitochondria and the development of Lewy bodies [42,43]. Increasing GSH levels in the brain is a possible therapy for PD, and multiple experimental investigations have shown this strategy works [44,45]. We discovered that *B. breve* CCFM1067 intake enhances the antioxidant capacity of the central nervous system, which helped PD mice reduce superoxide toxicity and alleviate oxidative stress. However, additional research is required to determine the precise mechanism by which *B. breve* CCFM1067 improves antioxidant levels in the brain.

Neuroinflammation is the primary cause of PD [46]. It accelerates the progression of PD by activating the brain microglia, which in turn generates pro-inflammatory substances through TLR4 receptors under the stimulation of MPP+ [47]. In our study, the brain tissue samples from MPTP-induced animals exhibited increased expression of both microglia (Iba-1) and astrocytes (GFAP). In contrast, mice administered *B. breve* CCFM1067 showed reduced MPTP-induced glial reactivity (Figure 2). Upregulated NLRP3 expression may enhance caspase-1-mediated inflammatory cascades and further stimulate the production of downstream inflammatory molecules, including IL-1β, IL-6, and TNF-α, all of which lead to an inflammatory response in a variety of neurological diseases [48,49]. In addition, in the postmortem brain, serum, and cerebrospinal fluid of patients with PD, inflammatory cytokines such as IL-1β, TNF-α, and IL-6 have been identified at excessively high concentrations [50,51]. This suggests that neuroinflammation plays a crucial role in the progression of neurodegenerative diseases [52]. According to our findings, the amount of IL-10 produced in mice with PD was drastically reduced, but the expression levels of IL-6, IL-1β, and TNF-α were markedly elevated (Figure 3). Moreover, our findings also showed that treatment with the *B. breve* CCFM1067 strain was able to reduce these inflammatory reactions and, of note, was better than L-DOPA in suppressing the expression of pro-inflammatory cytokines (Figure 3).

Intestinal inflammation is a crucial contributor to PD development [53,54,55,56]. Systemic inflammation induced by intestinal inflammation and barrier disruption is key in gut–brain communication [57,58,59]. Therefore, we examined the levels of three crucial cytokines (TNF-α, IL-1β, and IL-6) in the colon. We found that MPTP-induced PD animals exhibited intestinal inflammation with changes in inflammatory factor gene expression, consistent with the results in brain tissues (Figure 3). When *B. breve* CCFM1067 was administered orally, the level of colonic inflammation was much lower, indicating that the probiotic activity of *B. breve* CCFM1067 may assist in decreasing intestinal inflammation. According to previous studies, systemic inflammation may disrupt the BBB, allowing pro-inflammatory molecules (such as cytokines and LPS) to enter the CNS [60,61,62]. Tight junction proteins, such as ZO-1 and occludin, contribute significantly to the maintenance of the intestinal barrier, which aids in protecting immune cells underneath the intestinal mucosa from invading infections [54,63,64]. Our findings further demonstrate that the intestinal epidermal mucosa in the colon of MPTP-induced PD animals had an enhanced permeability, and that intervention with *B. breve* CCFM1067 significantly alleviated this change (Figure 3). Therefore, our data imply that intestinal barrier disruption caused by dysbiosis of the gut microbiota resulted in a pro-inflammatory cytokine leak, causing systemic inflammation. However, treatment with *B. breve* CCFM1067 reduced intestinal inflammation and prevented the development of PD. This was accomplished by improving the health of the intestinal barrier, preventing leakage of inflammatory molecules, and reducing inflammation in the brains of mice with PD.

A subsequent microbiota analysis revealed that the protective benefits of *B. breve* CCFM1067 could be mediated by the restoration of the normal microbiome. The α- and β-diversity data indicated that the microbial community of *B. breve* CCFM1067 was comparable to that of the control group (Figure 4). According to previous studies, MPTP induction may increase the occurrence of certain pathogenic bacteria and significantly impair the antioxidant and anti-inflammatory capabilities of the body [7,65], corroborating our findings. According to our research, MPTP intervention drastically changed the intestinal microbiota of PD mice (Figure 5), increasing *Bacteroides*, *Escherichia-Shigella*, and *Dubosiella*, and decreasing *Akkermansia* (Figure 5), which is consistent with the findings of previous studies [47,66,67]. In addition, treatment with *B. breve* CCFM1067 restored the altered levels of these bacteria; nevertheless, the MPTP-induced alteration of several taxa, most of which were members of the Bacteroidetes phylum, was irreversible. Interestingly, we discovered that the abundance of the *Bifidobacterium* genus increased after *B. breve* CCFM1067 administration, but there was no discernible variation in the genus after MPTP therapy. In the current study, pro-inflammatory cytokine levels were increased in the striatum and colon of MPTP-challenged mice but decreased after *B. breve* CCFM1067 administration. This indicates that *B. breve* CCFM1067 treatment may reduce inflammation and restore BBB integrity damaged by MPTP intoxication. This was further confirmed by the expression of three major tight junction proteins detected in the striatum and colon. It is worth noting that the correlation analysis revealed that the relative abundance of *Bifidobacterium* was substantially positively linked with relative ZO-1 expression in the striatum and colon. Meanwhile, our predicted metagenomic study showed MPTP-induced modifications in intestinal microbiota functions, specifically lowered “Biosynthesis of terpenoids and steroids,” “Sesquiterpenoid and triterpenoid biosynthesis,” “Steroid biosynthesis,” and “Carotenoid biosynthesis” (Figure 6), but these alterations were reversed by *B. breve* CCFM1067. In conclusion, we found that the ingestion of *B. breve* CCFM1067 after MPTP treatment led to variations in microbial function and restored the MPTP-induced alterations on multiple metabolic levels. Further research is required to ascertain the changes in the *Bifidobacterium* genus in PD after MPTP treatment, as this probiotic species is widely recognized for its anti-inflammatory benefits.

Furthermore, we found that MPTP-treated mice presented elevated levels of Enterobacteriaceae (Figure 5), which is thought to be implicated in both PD and AD [68]. Intriguingly, our findings revealed a greater number of *Escherichia-Shigella* in PD mouse feces, which significantly decreased after oral administration of *B. breve* CCFM1067. In our study, the *Escherichia-Shigella* genus was negatively correlated with DA, 5-HT, and DOPAC levels in the striatum, and positively correlated with movement disorder-related indicators. The *Escherichia-Shigella* genus is inversely correlated with illness duration, as reported by Qian et al. [69]. Chen et al. demonstrated that fisetin ameliorated the disorder by decreasing the number of *Escherichia-Shigella* in the intestines of PD mice [70]. However, our results demonstrate that *B. breve* CCFM1067 alleviated the MPTP-induced increase in Enterobacteriaceae. Both the data presented by Petrov et al. [71] and our own analysis show that the abundance of *Akkermansia* in PD substantially decreased. Additionally, we discovered that the abundance of the genus *Akkermansia* increased with *B. breve* CCFM1067 treatment. It has been widely assessed that *Akkermansia muciniphila*, which is one of the most numerous bacteria in the gut microbiota, has a role in metabolic illnesses, including obesity [72] and diabetes [73]. This bacterium has been heralded as the “next generation” of probiotics [74]. Although its mode of action has not been completely elucidated, mounting data suggest that, through the gut–brain axis, *A. muciniphila* is crucial for proper brain function and has therapeutic potential in several neuropsychiatric diseases [75,76]. In our study, we found that the abundance of *Dobusiella* in MPTP mice treated with *B. brev* CCFM1067 was comparable to that in the control group. Overall, our findings show that supplementing mice with *B. breve* CCFM1067 may reduce the symptoms of MPTP-induced PD by restoring a healthy gut microbial population.

According to reports, SCFAs generated by bacterial fermentation in the colon serve as essential mediators for the gut microbiota to regulate gut–brain communication [77]. SCFAs play an important role in human neurological illnesses because of their anti-inflammatory properties and their capacity to strengthen the BBB [78,79]. In addition, SCFAs can act as neuromodulators by regulating the synthesis of neurotransmitters (especially 5-HT) [80] as well as the expression of their receptors, thus providing neuroprotection [81]. The present results also show that PD mice treated with *B. breve* CCFM1067 exhibited substantial alterations in the gut microbial composition and a considerable increase in fecal SCFA content. Sampson et al. showed that microbiota-derived SCFAs are crucial for α-synuclein-mediated neuroinflammation, glial cell hyperactivation, and motor symptoms [10]. In our study, the increase in SCFAs may be one of the explanations for the anti-inflammatory action of *B. breve* CCFM1067 on the gut or brain of PD mice.

## 5. Conclusions

In summary, our study demonstrates that the probiotic *B. breve* CCFM1067 can exert neuroprotective effects on dopaminergic neurons in a subacute PD mouse model obtained via intraperitoneal injection of MPTP. We also showed that *B. breve* CCFM1067 improved the dyskinesia, death of dopaminergic neurons, and decrease in neurotransmitters caused by MPTP in mice. The possible mechanism by which *B. breve* CCFM1067 exerts neuroprotective effects in PD mice is through the increase in neurotrophic factor and SCFA levels, and the decrease in glial hyperactivation, resistance to oxidative stress injury, inflammatory response, and gut microbiota dysregulation, thus preventing dopaminergic neuron loss in the SN. Therefore, this study has preliminarily identified a new probiotic strain of *B. breve* CCFM1067, with promising applications as an effective oral supplement for PD prevention and therapy by influencing the gut–brain axis.

## Data Availability

The data sets generated during and/or analyzed during the current study are either shown in the manuscript and Supplementary Material or available from the corresponding author on reasonable request.

## Supplementary Materials

The following supporting information can be downloaded at: https://www.mdpi.com/article/10.3390/nu14214678/s1, Table S1: Primers information used for qRT-PCR; Figure S1: Iba-1, GFAP, BDNF and GDNF levels in the striatum; Figure S2: TNF-α, IL-1β, IL-6 and IL-10 levels in the striatum and colon; Figure S3: ZO-1, Occludin, and Claudin-1 levels in the striatum and colon; Figure S4: The differences in gut microbiota among the Control, MPTP, L-DOPA, and B. breve CCFM1067 groups at the genus level; Figure S5: Differences in predicted functional between L-DOPA and MPTP groups at the KEGG pathways at level 3; Figure S6: The relationships among 32 biomarkers of PD and 21 differential genera were estimated using Spearman correlation analysis.

## Abbreviations

α-SYN	α-synuclein
BBB	Blood–brain barrier
BDNF	Brain-derived neurotrophic factor
CAT	Catalase
CNS	Central nervous system
DA	Dopamine
DAB	3,3′-diaminobenzidine
DOPAC	3,4-dihydroxyphenylacetic acid
ELISA	Enzyme-linked immunosorbent assay
ENS	Enteric nervous system
GAPDH	Glyceraldehyde-3-phosphate dehydrogenase
GDNF	Glial cell line-derived neurotrophic factor
GFAP	Glial fibrillary acidic protein
GSH	Glutathione
HPLC	High Performance Liquid Chromatography
HVA	Homovanillic acid
Iba1	Ionized calcium binding adapter molecule 1
IL-1β	Interleukin-1β
IL-6	Interleukin-6
IL-10	Interleukin-10
IHC	Immunohistochemistry
LDA	Linear discriminant analysis
L-DOPA	Levodopa
LPS	Lipopolysaccharide
MGBA	Microbiota–gut–brain axis
MPTP	1-methyl-4-phenyl-1,2,3,6-tetrahydropyridine
MRS	De Man Rogosa Sharpe
NBT	Narrow-beam test
OUT	Operational taxonomic unit
OFT	Open field test
PCA	Principal component analysis
PCoA	Principal coordinate analysis
PD	Parkinson’s disease
perMANOVA	Permutational multivariate analysis of variance
PICRUSt	Phylogenetic Investigation of Communities by Reconstruction of Unobserved States
PT	Pole test
QIIME	Quantitative Insights into Microbial Ecology
qRT-PCR	Quantitative real-time polymerase chain reaction assay
ROS	Reactive oxygen species
RTR	Rotarod test
SCFAs	Short-chain fatty acids
SEM	Standard error of mean
SN	Substantia nigra
SOD	Superoxide dismutase
TNF-α	Tumor necrosis factor-α
TH	Tyrosine hydroxylase
TrkB	Tyrosine Kinase receptor B
ZO-1	Zonula occludens-1
5-HT	5-hydroxytryptamine
5-HIAA	5-hydroxyindoleacetic acid
