# Supplementary material for: Neuroprotective Effects of Bifidobacterium breve CCFM1067 in MPTP-Induced Mouse Models of Parkinson’s Disease
Source: Nutrients. 2022 Nov 4;14(21):4678. doi: 10.3390/nu14214678 (PMC9655354; doi:10.3390/nu14214678)
Supplement: Supplementary file 1 [file nutrients-14-04678-s001.zip › Supplementary.pdf]

Supplementary materials

1. Methods and materials

1.1 Strain and biological origin

Bifidobacterium breve CCFM1067 is stored at the Guangdong Microbial Culture Collection Center (NO. 60703). This strain was isolated from the feces of a healthy adult Beijing man by our laboratory, which was obtained from the in-house Culture Collections of Food Microbiology, Jiangnan University (Wuxi, China). Cryopreserved *B. breve* CCFM1067 was awakened by anaerobic culturing in de Man Rogosa Sharpe (MRS) liquid medium (supplemented with 0.05% L-cysteine) and grown anaerobically at 37°C for 18 h. The activated B. breve CCFM1067 was further cultured anaerobically in MRS broth at 37°C for 18 h, then harvested by centrifugation at 6000 × g for 10 min. Bacterial pellets were resuspended in MRS broth supplemented with 30% glycerol to a final concentration of 5×109 colony-forming units per milliliter (CFU/mL), then aliquoted in freezer tubes and stored at −80°C. Before oral administration, the cryopreserved B. breve CCFM1067 was thawed in a 37°C-water bath for 1 h, then centrifuged at 6000 × g for 10 min. The supernatants were removed, and the bacterial pellets were washed with saline and then centrifuged at 6000 × g for 10 min. The supernatants were then removed, and the bacterial pellets were re-suspended at an objective concentration with saline.

1.2 Behavioral tests for motor functions

The PT was used to evaluate agility and sensorimotor impairments (Ogawa et al., 1985). A 50-cm vertical pole (1-cm diameter) with a rough surface was placed in a cage. On each training day, the mice were first placed in the pole cage for environmental familiarization, then placed head-upward on the pole 15 cm above the cage floor 3 times, followed by 30 cm and 50 cm above (top of the pole) for training. On the test day, each mouse was placed head-upward on the top of the pole, and the times taken to turn around to head-down position (T-turn) and to descend to the floor (T-total) were recorded for 60 s. If the mouse fell or jumped off the pole during the 60 s, the test was repeated. If the mouse could not turn head downward or kept falling off the pole, both the T-turn and T-total times were recorded as 60 s. Each mouse was tested 3 times (with 1-minute intervals between tests), and the average time was taken.

The NBT was used to evaluate motor coordination and balance (Luong et al., 2011). The beam apparatus comprised a 50-cm beam with a 0.8-cm-wide flat surface. The beam was set 50 cm above the table top on two poles. A black box was placed at one end of the beam as the finish point. Mouse chow was placed in the black box to attract the mouse. On each training day, mice were placed in the black box for 5 min for environmental familiarization, then placed on the beam 5 cm away from the box beam to train them walk towards the box 3 times. This was followed by 15-, 30- and 50-cm beam-walk training. On the test day, each mouse was tested 3 times (with 1-minute intervals between tests). The time required to cross the beam and enter the finish was recorded until completion or a maximum of 60 s. The test was redone if the mouse fell or turned back to the starting point halfway. If the mouse could not walk to the finish within 60 s or kept falling off the beam, a total time of 60 s was recorded. The average time from the 3 trials was taken.

The RTR was used to assess motor coordination (Shiotsuki et al., 2010). The RT-01 mouse rotarod (SINGA, Taiwan) containing automatic timers and falling sensors was used for both the training period and formal test. All mice were first trained at 10 rpm rotary speed for 180 s 3 times on day 20, followed by 20 rpm rotary speed on day 21 and 30 rpm rotary speed on day 22. On the test day, each mouse was

tested at 30 rpm rotary speed for 180 s 3 times (1-minute intervals between tests); the latency time to fall was recorded, and the average time was taken.

The open field consisted of a square arena (50 cm×50 cm) enclosed by continuous opaque walls made of plexiglass. Mice were placed into the middle of the area facing the wall, locomotor activity was monitored for 10 minutes, the time spent in the center and the ratio of distance traveled in the center was measured.

1.3 Quantitative real-time PCR (qRT-PCR)

Table S1. Primers information used for qRT-PCR

Primer	GenBank Accession	Sequence (5'-3')	Location	Amplicon Size (bp)
TH	NM_009377	CCCAAGGGCTTCAGAAGAG	65-83	106
		GGGCATCCTCGATGAGACT	170-152	
Aif1(*Iba1*)	NM_001361501	TCTGCCGTCCAAACTTGAAGCC	443-464	138
		CTCTTCAGCTCTAGGTGGGTCT	580-559	
Gfap	NM_001131020	AAGCAGATGAAGCCACCCTG	3940-3959	625
		GTCTGCACGGGAATGGTGAT	4564-4545	
Bdnf	NM_001316310	GAAGAGCTGCTGGATGAGGAC	517-537	332
		TTCAGTTGGCCTTTTGATACC	848-828	
Gdnf	NM_001301357	TCACCAGATAAACAAGCGG	297-315	175
		TTTCATAGCCCAAACCCAA	471-453	
IL-6	NM_001314054	TTCCATCCAGTTGCCTTCT	103-121	173
		CTCATTTCCACGATTTCCC	275-257	
Tnf-α	NM_013693	CCAGACCCTCACACTCAGA	392-410	187
		GACAAGGTACAACCCATCG	578-560	
IL-1β	NM_008361	CCTGTGTCTTTCCCGTGGA	316-334	357
		GGGTGTGCCGTCTTTCATT	672-654	
IL-10	NM_010548	GCTCTTACTGACTGGCATGAG	220-240	105
		CGCAGCTCTAGGAGCATGTG	324-305	
Tjp1(*ZO-1*)	XM_036152895	GGGAGGGTCAAATGAAGACA	6366-6385	145
		GGCATTCCTGCTGGTTACAT	6510-6491	
Occluldin	NM_001360538	ATAATGGGAGTGAACCCGACG	724-744	187
		CCACGATAATCATGAACCCCA	910-890	
Claudin-1	NM_016674	TGGATGGCTGTCATTGGGG	562-580	127
		ACCTGGCATTGATGGGGGT	688-670	
Gapdh	XM_036165840	ACCACAGTCCATGCCATCAC	946-965	452
		TCCACCACCCTGTTGCTGTA	1397-1378	

1.4 Metagenomic sequencing

Briefly, total DNA was extracted from the fresh stool samples first, according to the instructions laid out in the Fast DNA Stool Kit (MP Biomedicals, CA, USA). Then amplicons of the V3-V4 region of 16S rRNA were generated by PCR reaction using universal primers (341F/806R) and purified using TIANgel Mini Purification Kit (TIANGEN, Beijing, China). DNA was quantified and pooled in equal concentrations following the instructions of the Qubit dsDNA Assay Kit (Life Technologies, CA, USA). Samples were barcoded and finally paired-end sequenced on the Illumina MiSeq PE300 platform following the manufacturer’s protocol.

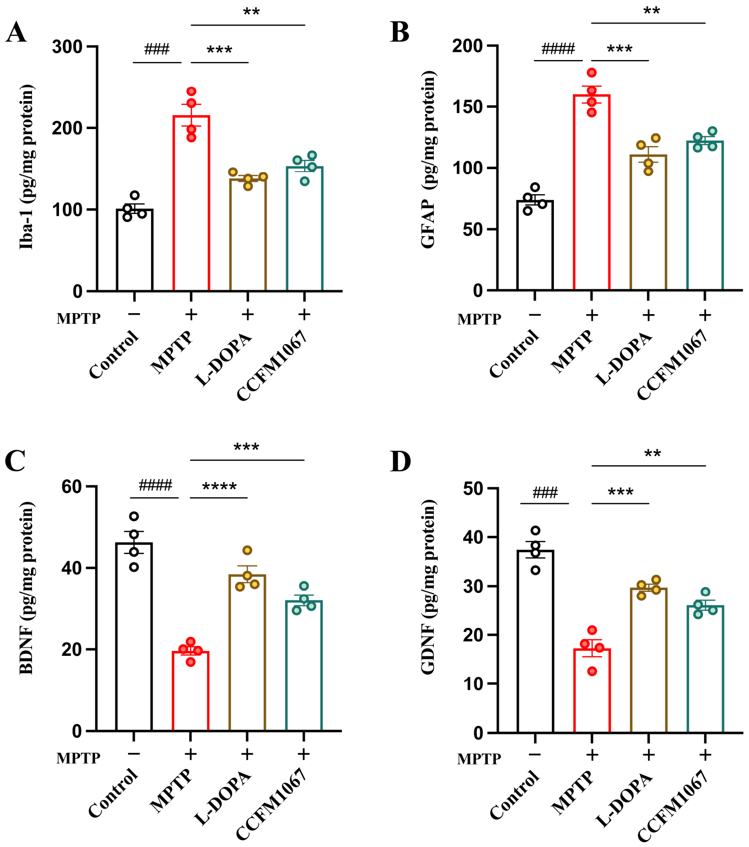

Figure S1. Iba-1, GFAP, BDNF and GDNF levels in the striatum. Proinflammatory cytokines levels were analyzed via ELISA and normalized to the protein concentrations in the brain tissues (pg/mg protein). Data are expressed as means ± SEM and analyzed via one-way ANOVA with Tukey's post hoc test. $^{###}p < 0.001$, $^{####}p < 0.0001$, compared with the Control group; $^{**}p < 0.01$, $^{***}p < 0.001$, $^{****}p < 0.001$, compared with the MPTP group (n = 4/group).

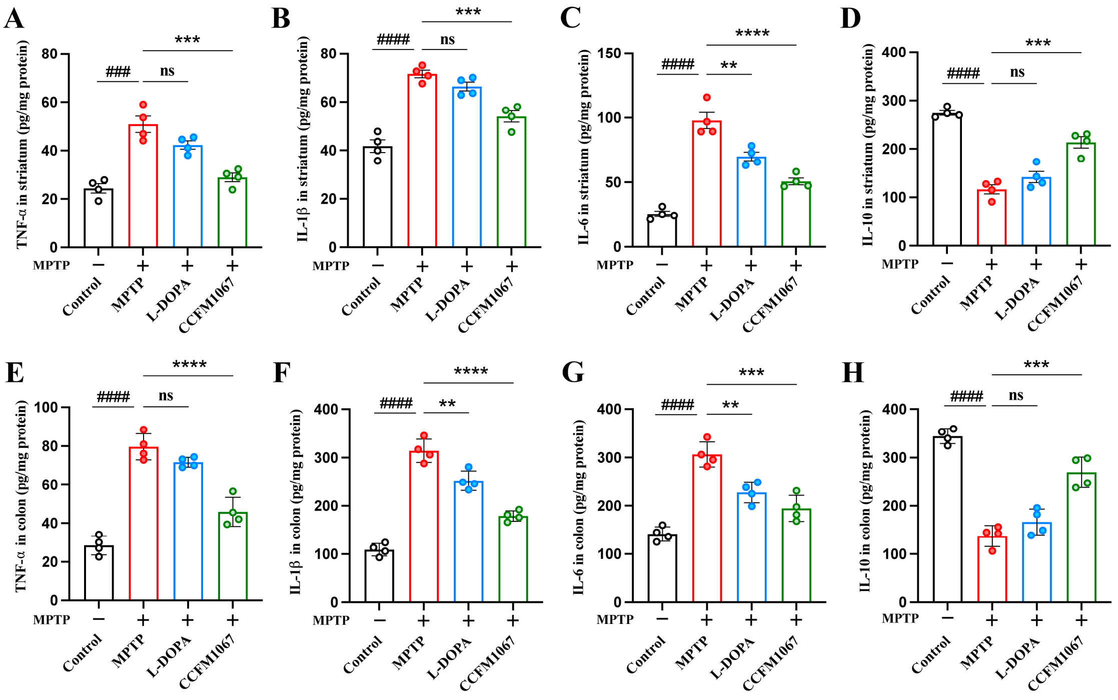

Figure S2. TNF-α, IL-1β, IL-6 and IL-10 levels in the striatum and colon. Proinflammatory cytokines levels were analyzed via ELISA and normalized to the protein concentrations in the brain tissues (pg/mg protein). Data are expressed as means ± SEM and analyzed via one-way ANOVA with Tukey's post hoc test. $^{\#\#\#}p < 0.001$, $^{\#\#\#\#}p < 0.0001$, compared with the Control group; $^{**}p < 0.01$, $^{***}p < 0.001$, $^{****}p < 0.001$, compared with the MPTP group (n = 4/group).

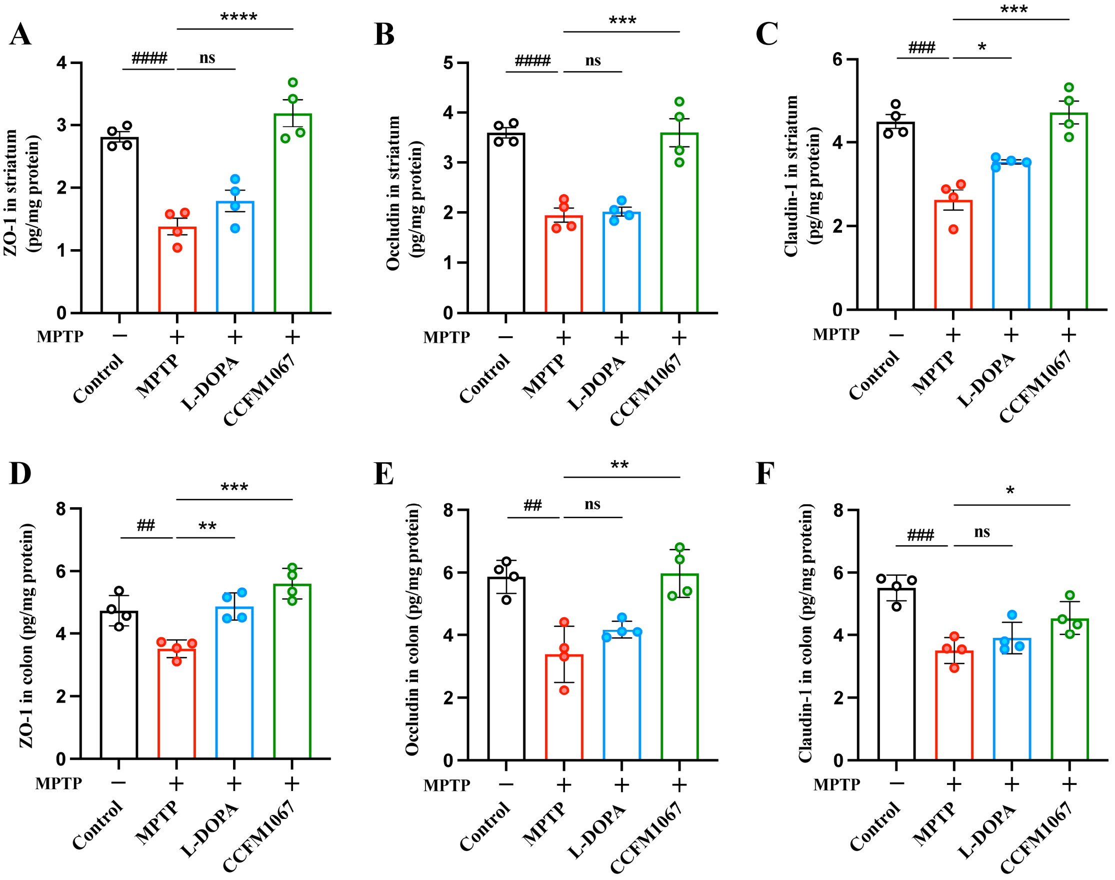

Figure S3. ZO-1, Occludin, and Claudin-1 levels in the striatum and colon. Proinflammatory cytokines levels were analyzed via ELISA and normalized to the protein concentrations in the brain tissues (pg/mg protein). Data are expressed as means ± SEM and analyzed via one-way ANOVA with Tukey's post hoc test. $^{\#\#}p < 0.05$, $^{\#\#\#}p < 0.001$, $^{\#\#\#\#}p < 0.0001$, compared with the Control group; $^{*}p < 0.05$, $^{**}p < 0.01$, $^{***}p < 0.001$, $^{****}p < 0.001$, compared with the MPTP group (n = 4/group).

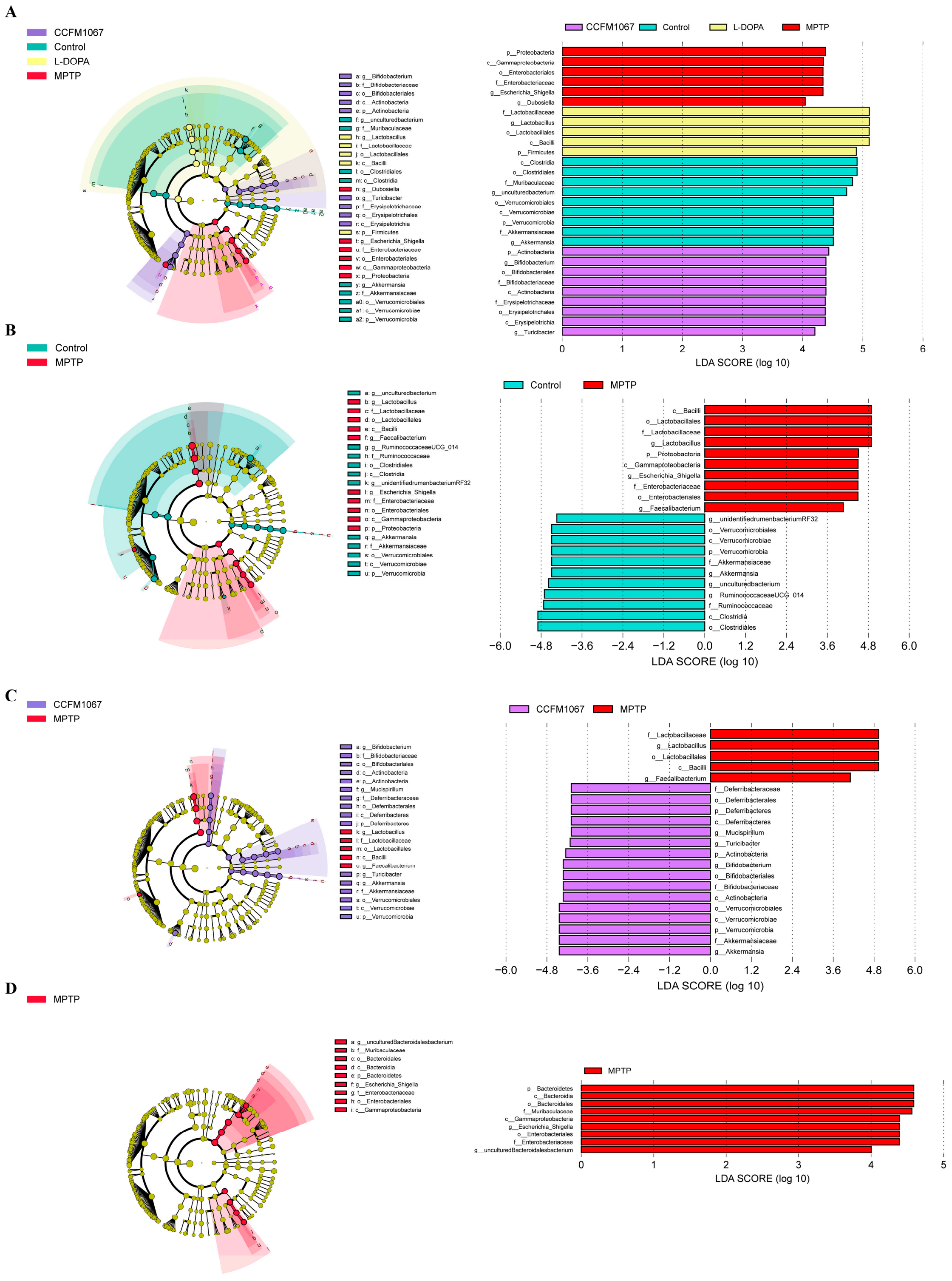

Figure S4. The differences in gut microbiota among the Control, MPTP, L-DOPA, and *B. breve* CCFM1067 groups at the genus level (A) Linear discriminant analysis (LDA) effect size (LEfSe). LEfSe analysis among Control, MPTP, L-DOPA, and *B. breve* CCFM1067 groups. The differentially abundant taxa in the taxonomic tree are shown in the cladogram in different colors. Differential taxa are labeled with tags and annotated in the right panel. The LDA scores greater than 4.0 for the significantly differentially abundant bacteria are displayed in the histogram with different colors. (B) LEfSe analysis

among the Control and MPTP groups. (C) LEfSe analysis among the MPTP and *B. breve* CCFM1067 groups. (D) LEfSe analysis among the MPTP and L-DOPA groups.

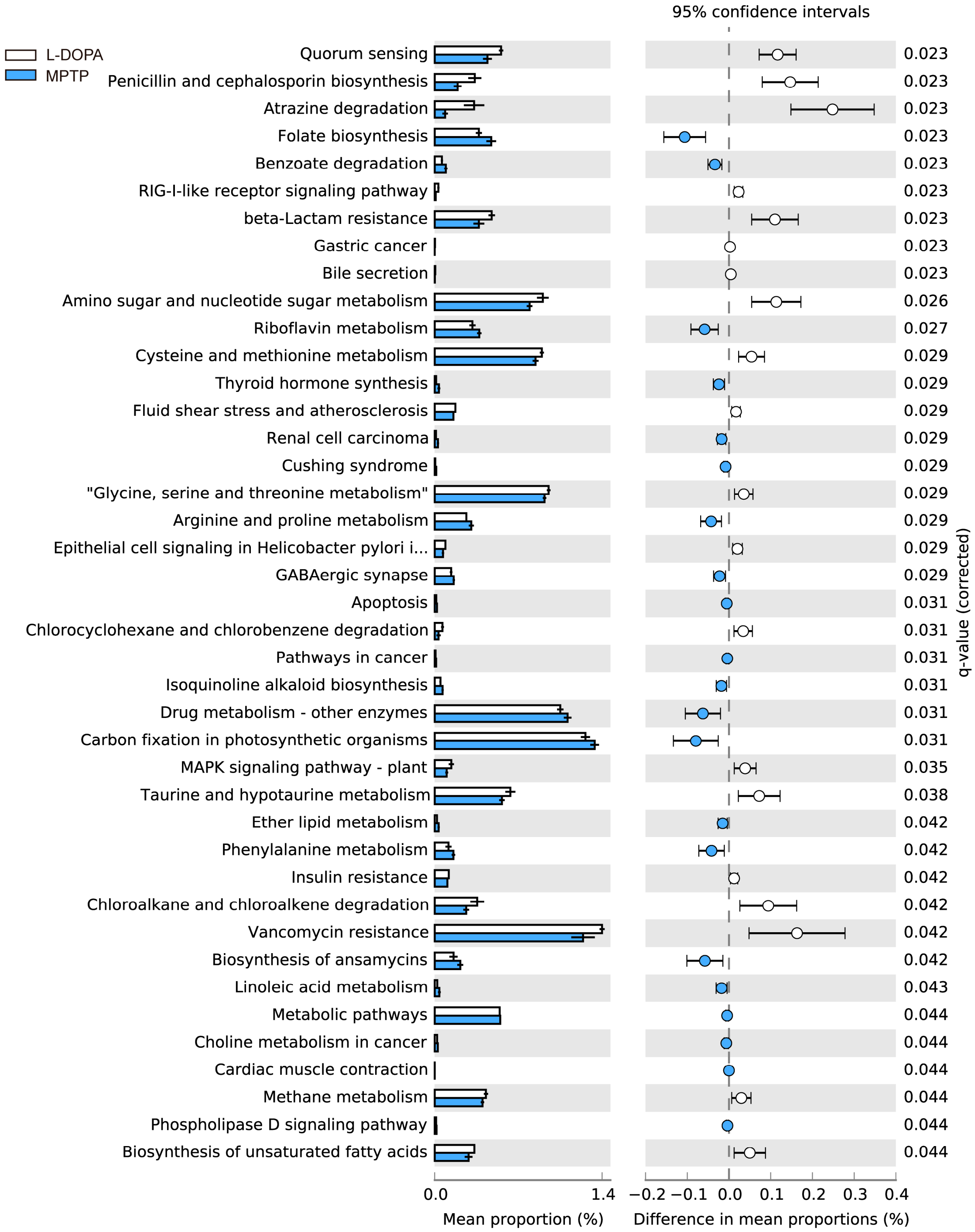

Figure S5. Differences in predicted functional between L-DOPA and MPTP groups at the KEGG pathways at level 3 (Welch's t-test, two-sided, Storey FDR $q < 0.05$)

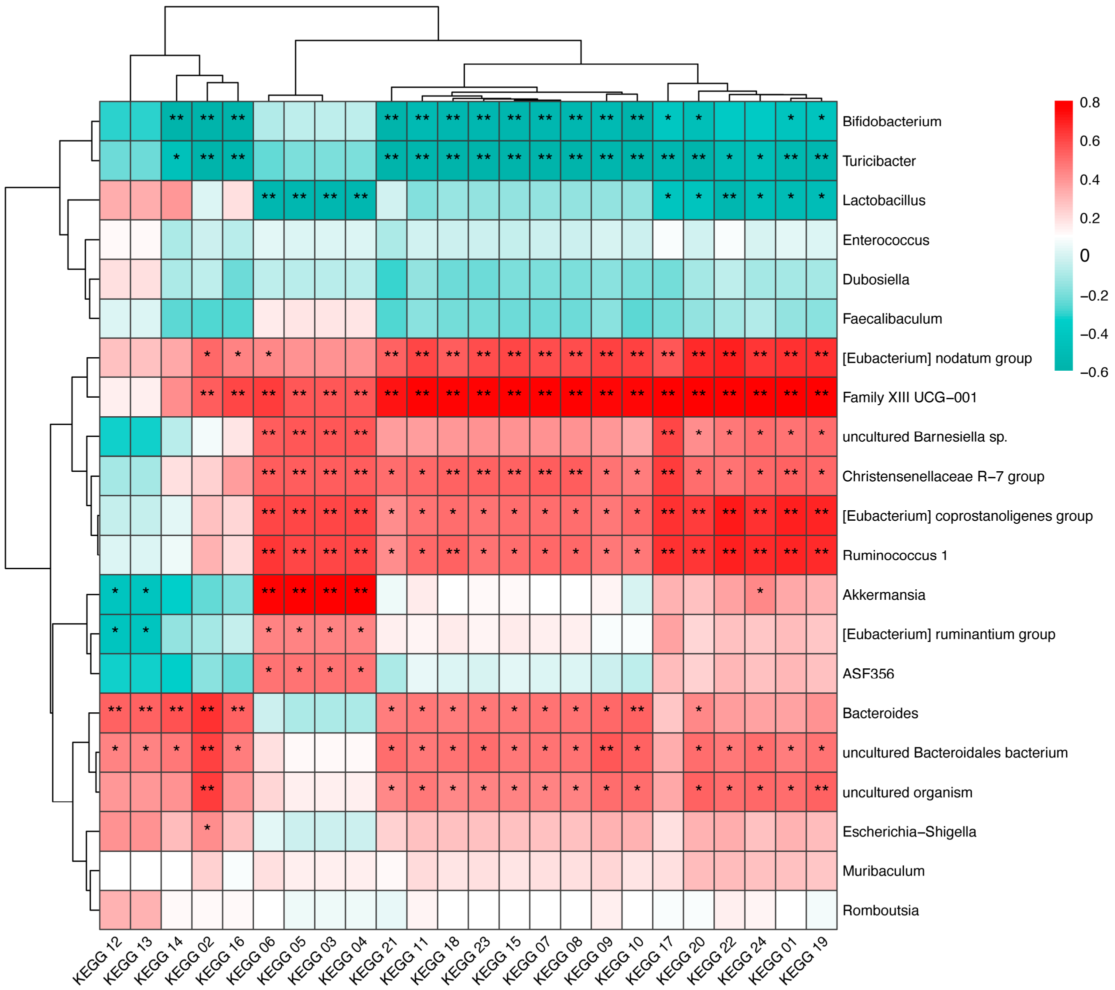

KEGG 01: Phenazine biosynthesis
KEGG 02: Benzoate degradation
KEGG 03: Biosynthesis of terpenoids and steroids
KEGG 04: Sesquiterpenoid and triterpenoid biosynthesis
KEGG 05: Steroid biosynthesis
KEGG 06: Carotenoid biosynthesis
KEGG 07: Human papillomavirus infection
KEGG 08: Viral carcinogenesis
KEGG 09: Drug metabolism-other enzymes
KEGG 10: Butanoate metabolism
KEGG 11: Pentose phosphate pathway
KEGG 12: Renal cell carcinoma
KEGG 13: Cushing syndrome
KEGG 14: Synthesis and degradation of ketone bodies
KEGG 15: Type II diabetes mellitus
KEGG 16: Aminobenzoate degradation
KEGG 17: AMPK signaling pathway
KEGG 18: Alzheimer disease
KEGG 19: Phenylpropanoid biosynthesis
KEGG 20: Phenylalanine, tyrosine and tryptophan biosynthesis
KEGG 21: Central carbon metabolism in cancer
KEGG 22: PI3K-Akt signaling pathway
KEGG 23: Ribosome biogenesis in eukaryotes
KEGG 24: Glycosaminoglycan degradation

Figure S6. The relationships among 32 biomarkers of PD and 21 differential genera were estimated using Spearman correlation analysis. Color intensity represents the magnitude of correlation. Red, positive correlations; green, negative correlations. * p <0.05; ** p <0.01; *** p <0.001.
